# Expert interpretation of bar and line graphs: the role of graphicacy in reducing the effect of graph format

**DOI:** 10.3389/fpsyg.2015.01673

**Published:** 2015-10-30

**Authors:** David Peebles, Nadia Ali

**Affiliations:** Applied Cognition and Cognitive Engineering Group, Centre for Applied Psychological and Health Research, Department of Behavioural and Social Sciences, University of HuddersfieldHuddersfield, UK

**Keywords:** expertise, graph comprehension

## Abstract

The distinction between informational and computational equivalence of representations, first articulated by Larkin and Simon (1987) has been a fundamental principle in the analysis of diagrammatic reasoning which has been supported empirically on numerous occasions. We present an experiment that investigates this principle in relation to the performance of expert graph users of 2 × 2 “interaction” bar and line graphs. The study sought to determine whether expert interpretation is affected by graph format in the same way that novice interpretations are. The findings revealed that, unlike novices—and contrary to the assumptions of several graph comprehension models—experts' performance was the same for both graph formats, with their interpretation of bar graphs being no worse than that for line graphs. We discuss the implications of the study for guidelines for presenting such data and for models of expert graph comprehension.

## 1. Introduction

A widely established finding in the diagrammatic reasoning literature is that the interpretation and comprehension of information can be significantly affected by the format of its representation. The phenomenon of two graphical representations of the same information resulting in very different behavior has been reported on numerous occasions (e.g., Zacks and Tversky, 1999; Peebles and Cheng, 2003; Kosslyn, 2006; Peebles, 2008) and is typically explained in terms of the distinction between *informational* and *computational* equivalence of representations (Larkin and Simon, 1987). According to this account, observed variation in behavior is due primarily to the fact that different graphical representations facilitate the use of different cognitive and perceptual operators.

**Figure 1:**
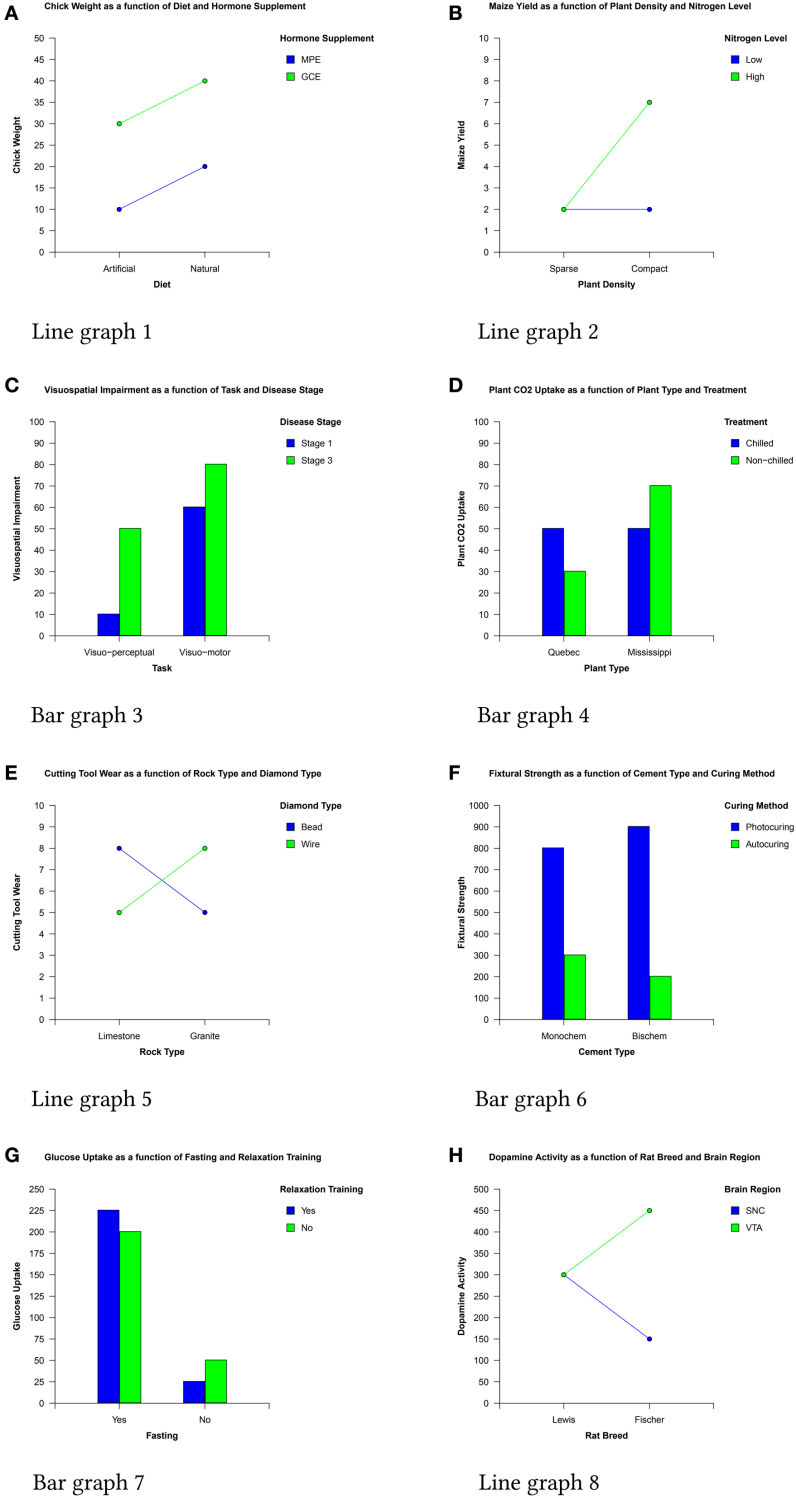
**The eight data sets used in the experiment**.

Take two widely used representations—bar and line graphs—as an example (see Figure 1). These two formats share a key structural feature; the graphical framework provided by the x and y axes, which defines the Cartesian coordinate system. It has been argued that this framework is an essential element of people's mental representation (or *schema*) of these graphs stored in long-term memory that acts as a visual cue for the stored mental representation which is then used to interpret the graph (Ratwani and Trafton, 2008).

Despite this common framework, the distinct features of bar and line graphs result in significant differences in their interpretation. Because lines bind plotted points into single objects, people encode them in terms of their slope (e.g., Simcox, 1983, reported by Pinker, 1990), interpret them as representing continuous changes on an ordinal or interval scale (Zacks and Tversky, 1999; Kosslyn, 2006), and are generally better at identifying trends and integrating data using line graphs (Schutz, 1961).

This is not the case for bar graphs however. Because data points are represented by individual separate bars, they are more likely to be encoded in terms of their height, interpreted as representing distinct values on a nominal scale, and are therefore better for comparing and evaluating specific quantities (Culbertson and Powers, 1959; Zacks and Tversky, 1999).

In a series of experiments, we have investigated the effect of format on the interpretation of *interaction* graphs (Peebles and Ali, 2009; Ali and Peebles, 2011, 2013). Interaction graphs (in both bar and line form) are widely used in the analysis and interpretation of data from factorial design experiments, a complex skill that requires detailed knowledge and substantial practice to do correctly. The pervasiveness of factorial research designs in science, engineering, business, and medicine places them centrally in the curricula of these disciplines and they are employed and studied by many thousands of people globally.

The production and interpretation of graphical representations of statistical analysis results is an important element of training to use factorial designs. For example, the simplest, most common, and often earliest encountered design is the 2 × 2 factorial design which investigates the effects and interactions of two factors (each of which has two levels) on a dependent variable. Statistical analysis of this design typically results in a 2 × 2 matrix of the mean dependent variable values corresponding to the pairwise combinations of each factor's levels and graphs of this matrix (examples of which are shown in Figure 1) are frequently produced to help interpret the data.

In our studies we have investigated how the different graphical features of bar and line graphs affect how people interpret data due to the operation of different Gestalt laws of perceptual organization (Wertheimer, 1938). The Gestalt principles of *proximity, similarity, connectedness, continuity*, and *common fate* determine how graphical features are grouped by the human visual system to form coherent wholes and play a crucial role in determining how data are interpreted and the nature of the mental representations that users generate when using graphs (e.g., Kosslyn, 1989; Pinker, 1990; Shah et al., 1999).

For example, the x variable values in bar graphs are grouped together on the x axis and, as a result of the Gestalt principle of *proximity* (Wertheimer, 1938) each cluster of bars forms a separate visual chunk (Peebles and Ali, 2009). People then use these chunks as the basis for comparing the levels of the legend variable (e.g., in Figure 1D a user may say “if Quebec plants are not chilled, they take up less CO_2_ than when they are chilled, but if Mississippi plants are not chilled, they take up more CO_2_ than when they're chilled”).

In the case of line graphs however, data points are connected by lines which, by the Gestalt principle of *connectedness* (Palmer and Rock, 1994), form individual visual chunks (Peebles and Ali, 2009). People rapidly identify these chunks, access the associated label in the legend by color (via the Gestalt law of *similarity*) and then use them as the basis for comparing the levels of the x variable (e.g., in Figure 1E a user may say “for bead diamonds, limestone produces more cutting tool wear than granite, but for wire diamonds the opposite is true”).

Because of this, people are more likely to describe relationships as a function of the variable plotted on the x axis when using bar graphs but more likely to describe them as a function of the legend variable when using line graphs (Peebles and Ali, 2009; Shah and Freedman, 2009; Ali and Peebles, 2013).

### 1.1. The relationship between graph format and graphical literacy

The effect of graph format on interpretation is particularly pronounced and deleterious for inexperienced users. In our experiments we have demonstrated that non-expert users perform significantly worse using line graphs than when using equivalent bar graphs (Peebles and Ali, 2009; Ali and Peebles, 2011, 2013). Our studies revealed that non-expert line graph users consistently ignore or are unable to interpret the variable plotted on the x axis.

The reason for this is that bar graphs allow the operation of two Gestalt principles to take place which results in a more balanced representation of the data. In bar graphs, as a result of the Gestalt principle of *proximity* (Wertheimer, 1938), each cluster of bars forms a separate visual chunk anchored to the x axis. When people attend to these chunks, they are able to identify the nearby *x* value label quickly and easily and associate the bars with the variable plotted on the x axis. In addition, the bars are also usually colored or shaded, with the legend containing similar patches next to the level labels of the z variable. According to the Gestalt principle of *similarity*, this shared color or shade allows users to associate each bar with its associated level rapidly and easily. The two principles combined ensure that users attend to both variables equally.

In line graphs however, data points are usually represented by colored shapes connected by similarly colored lines. According to the Gestalt principle of *connectedness* (Palmer and Rock, 1994), each line with its two end points forms an individual visual chunk. As in the case of the bar graphs, line graph users are able to associate each line with a level of the legend variable by shared color and the Gestalt principle of similarity. Unlike the bar graphs however, there is no equivalent perceptual grouping process available in the line graphs to facilitate the association between the points at the ends of the lines and the variable values on the x axis. Although points and labels may be associated by vertical alignment, our studies showed that this association is not sufficient to counterbalance the color-matching process, most likely because perceiving the line as the primary representational feature impairs users' ability to differentiate the points from the line.

Based on these findings and our understanding of how Gestalt principles operate, we developed a modified version of the line graph that produces a more balanced representation and which significantly reduces the biases and errors found in novices' interpretations (Ali and Peebles, 2013).

Our research demonstrated how the graphical and representational features of different graphs can strongly affect the performance of individuals with relatively little experience. However, a number of intriguing questions remain about how expert users interpret data using both graph formats. Specifically, it would be valuable to know precisely what knowledge and cognitive processes underlie expert performance and to determine to what extent (if at all) experts' interpretations are affected by the graph type used. If it is found that expert performance is largely unaffected by graph format then identifying the knowledge that determines this general skill will be useful to improve the training and instruction of novices. Conversely, if it is found that experts' abilities do differ between bar an line graphs and are more attuned to a specific format, then this will also be valuable in evaluating the appropriateness of the two graph types for different tasks and classes of user.

In relation to graph interpretation, expertise consists of two core elements; (a) knowledge of the domain and the methods by which the information in the graph was obtained or created, and (b) general graphical literacy, or “graphicacy” (Friel and Bright, 1996; Friel et al., 2001; Shah and Freedman, 2009). The latter consists of knowledge of how classes of diagrams work, including the properties of coordinate systems (e.g., the principle that the distance between two graphical elements encodes the magnitude of a relationship between the concepts represented by those elements), and the typical allocation of the dependent and independent variables to the axes and legend. This knowledge allows users with high levels of graphical literacy to mentally manipulate and transform the data in the graph (for example by knowing how to identify or compute the mean value of a set of points) to generate inferences that non-expert users could not.

### 1.2. Pattern recognition and expert graph comprehension

Another key aspect of expert graph use is the ability to recognize and interpret common patterns, a characteristic of expert performance found in many domains, from chess playing (Chase and Simon, 1973; De Groot, 1978), medical diagnosis (Norman et al., 2007), to geometry problem solving (Koedinger and Anderson, 1990).

In interaction graphs, a small number of quite distinct and relatively common patterns exist which experts learn to identify rapidly, either through explicit instruction (e.g., Aron et al., 2006) or simply through repeated exposure. Four patterns indicating the existence (or otherwise) of interaction effects are particularly common and readily identified: the “crossover interaction” shown in Figure 1E, the “less than” or “greater than” pattern shown in Figure 1H, and a related “angle” pattern formed by a horizontal and a sloped line (Figure 1B). In contrast, parallel lines (e.g., Figure 1A) signal that there is no interaction between the IVs.

In addition to these interaction patterns, two patterns indicating substantial main effects can also be recognized by experts (and are often rapidly identified by novices due to their visual salience). These patterns are shown in Figure 1. The large gap between the mid-points of the two lines in Figure 1A shows a large main effect of the legend variable while the large difference between the mid-points of the two values representing each x axis level in Figure 1G reveals a large main effect of the x axis variable.

These two examples highlight an additional source of bottom-up, data-driven effects on interpretation not associated with the features of a specific graph format but which influences the patterns formed similarly in both graph formats. Specifically, the relative sizes of the main and interaction effects in a particular data set determine the patterns formed in the graph and the relative salience of the effects. It is possible that this could influence the order in which experts interpret effects as larger effects are represented by wide gaps between lines or bars which may be more perceptually salient than smaller gaps. The possibility that graph comprehension performance is determined by the interaction between the patterns formed by various relationships in the data and the size of those relationships will be discussed and investigated further below.

#### 1.2.1. A pattern recognition based cognitive model of expert graph comprehension

Following the novice study conducted by Ali and Peebles (2013), Peebles (2013) carried out a detailed cognitive task analysis of the comprehension of 2 × 2 interaction graphs to produce a cognitive model implemented in the ACT-R cognitive architecture (Anderson, 2007). The model is informed by foundational work on graphical perception (Cleveland and McGill, 1984) and includes a precise specification of the declarative and procedural knowledge required to produce a complete and accurate interpretation of 2 × 2 interaction graphs and a set of assumptions and hypotheses about the processes by which experts interpret them. Specifically, the model contains representations in long-term memory that associate individual patterns or visual indicators in the graph with particular interpretations. The model also contains strategies for visually scanning the graph (encoded as a set of production rules) as well as a set of production rules to identify patterns. When a pattern is identified, a chain of subsequent productions is triggered which obtains further information from the graph and declarative memory until an interpretation is produced. This process continues until all patterns have been identified and interpreted appropriately, and an accurate mental model of the state of affairs depicted in the graph has been generated.

The ACT-R model is able to produce a complete and accurate interpretation of any data presented in three-variable line graphs at the level of a human expert and can explain its interpretations in terms of the graphical patterns it uses^1^. As such it can be considered a form of expert system built within the constraints of a theoretically grounded cognitive architecture. It remains an open question however, to what extent the behavior of human experts conforms to this ideal model and if not, what constitutes and underlies sub-optimal performance.

It is also not clear to what extent the assumptions underlying the expert model apply equally to the comprehension of line and bar graphs. Although the model has only currently been applied to line graphs, the key information that the model encodes from the display is the set of x-y coordinate locations of the four data points and the distances between them. Therefore, the pattern matching rules used by the model do not rely on specific features of the line graph but are defined in relation to the patterns formed by the coordinate points. It would be trivial to present the model with a set of equivalent bar graphs and the model would predict no significant difference in behavior.

If empirical studies were to reveal however that experts do in fact behave differently with the two formats (or by the relative sizes of the various effects in a data set), then the assumptions of the model would have to be revised to incorporate these processes.

### 1.3. Aims of the study

As alluded to earlier, two contrasting hypotheses may be produced concerning the relationship between levels of graphical literacy and the interpretation of different graph types. The first is that users with high graphicacy should be affected less by graph format because they should be able to identify and mentally manipulate relevant information in the graph and generate appropriate inferences irrespective of the graphical features used to represent it (Pinker, 1990).

The second hypothesis is that experts' greater exposure to the different graph formats and their learning of common patterns creates a set of expectations about the functions and properties of each format. For example, expert users may develop the expectation that the function of line graphs is to display interactions while that of bar graphs is to present main effects (Kosslyn, 2006). This may bias experts' interpretations and result in experienced users being equally, if not more, affected by presentation format than non-experts.

Using a student sample divided into “high” and “low” graphicacy groups, Shah and Freedman (2009) examined these competing predictions and found that expectation did not influence interpretation in a straightforward way. Rather, they found that high graphicacy students were only influenced by format expectations when the graph depicted data from a known domain. Specifically, high graphicacy students were more likely to identify main effects in bar graphs only when the subject matter was familiar to them. When the domain was unfamiliar, there was no difference in performance between graph formats. The authors did find however that the identification of interactions from both high and low graphicacy participants was affected by graph format in the predicted way (i.e., more descriptions as a function of the x axis variable with bar graphs and more descriptions as a function of the legend variable with line graphs).

While it is unclear to what extent high graphicacy students can be considered experts, Shah and Freedman's experiment can be seen as providing at least tentative evidence that could challenge previous recommendations to use line graphs because of experts' ability to recognize interactions using common patterns created by the lines (e.g., Pinker, 1990; Kosslyn, 2006). Shah and Freedman found no effect of graph skill on interaction descriptions and while they did show that both high and low graphicacy participants were affected by graph format, they found no evidence that line graphs supported identification of interactions more than bar graphs in either group. It may be the case therefore, that once users have obtained a certain level of graphical literacy, they are able to apply their knowledge to override differences in Gestalt grouping or visual salience between graph types to interpret data appropriately whatever graph they use.

The experiment reported here aims to answer the questions raised in the above discussion by focusing more closely on the types of individuals we study. Unlike previous research in this area (including our own) that has predominantly used undergraduate students, we recruited participants from academic faculty in the areas of scientific psychology and cognitive science who have sufficient experience (either through teaching or research or a combination of both) of ANOVA designs to be considered expert users of interaction graphs.

The sample was representative of the range of expertise typically found in academia and ranged from early career researchers and assistant professors to full professors. Experience in the field at post-doctoral level ranged from a few years to decades. The sample was gathered from multiple centers and participants included British and international academics who could be considered experts in the field. Using this participant group, we aim to determine whether experts' interpretations of unfamiliar data differ depending upon whether the data is presented in bar or line graph form. In so doing we also aim to ascertain the relative effects of bottom-up and top-down processes (i.e., to determine the relative effects of user expectations and graphical features). This will allow us to quantify the amount of benefit, if any, that line graphs provide for expert users (as suggested by Kosslyn, 2006) and to determine whether this is outweighed by other factors (e.g., effect sizes in the data).

The second aim of this experiment is to determine whether the processes by which experts achieve their interpretations differ using the two graphs. Although it may be the case that experts are able to produce accurate and roughly equivalent interpretations of bar and line graphs, the processes by which they do so may be quite different and affected significantly by graphical features. Specifically, previous studies using non-expert samples have shown that graph format affects the order in which people interpret the graph; people typically interpret the legend variable before the x axis variable when using line graphs (Shah and Carpenter, 1995) but the opposite order when using bar graphs (Peebles and Ali, 2009). In addition, line graphs may facilitate pattern recognition processes that bar graphs do not which may lead to more rapid identification of interaction effects.

A third, related aim of the experiment is to determine whether interpretation order is affected significantly by the relative size (and as a result salience) of the patterns formed by the various relationships in the data.

By recording a range of behavioral measures such as the number of correct interpretations, the sequential order of interpretations, and task completion times, together with concurrent verbal protocols, we aim to construct detailed hypotheses relating to the processes underlying expert graph comprehension and to use the information obtained to evaluate the assumptions of the cognitive model, specifically the hypothesis that expert performance can be accounted for by a sequence of pattern recognition and knowledge retrieval processes.

Verbal protocol analysis is a technique widely used in cognitive science to obtain information about the processes being employed to perform tasks (Newell and Simon, 1972; Ericsson and Simon, 1984) which has successfully brought to light a wide range of phenomena including nonverbal reasoning (Carpenter et al., 1990), diagrammatic reasoning (Koedinger and Anderson, 1990), and graph comprehension (Shah et al., 1999). The “think aloud” method we employ in this study is one of the most commonly used techniques for obtaining verbal protocols and there is considerable empirical evidence that it is relatively unobtrusive and does not significantly affect cognitive processing (Crutcher, 1994; Fox et al., 2011).

Taken as a whole, the verbal protocol and other behavioral data will allow us to determine the extent to which experts' performance differs from the optimal predictions of the model and provide valuable information to inform revisions of the currently assumed mechanisms and processes.

## 2. Methods

### 2.1. Participants and design

The participants were 42 (11 female, 31 male) university faculty (i.e., assistant, associate, and full professors) or post-doctoral researchers in cognitive psychology or cognitive science. Forty were educated to PhD level while two were in the latter stages of working toward a PhD while being employed as university teaching fellows. Participants were gathered from three locations. The majority of participants were faculty specializing in cognitive psychology and quantitative research methods from the universities of Keele and Huddersfield in the UK. The remaining participants were cognitive scientists attending an international conference on cognitive modeling.

The experiment was an independent groups design with one between-subject variable: the type of diagram used (bar or line graph) and 21 participants were allocated to each condition using a random process.

### 2.2. Materials

The stimuli were 16 three-variable interaction graphs—eight line and eight bar—depicting a wide range of fictional content using variables taken from a variety of (non-psychology related) sources. Each of the eight data sets (shown in Figure 1) used to produce the graphs depicted the effects of two independent variables (IVs) on a dependant variable (DV) as would be produced by a 2 × 2 factorial research design.

The data sets were generated to create the main effects and interactions commonly encountered in these designs in a range of sizes. The y axis for all graphs started at zero and had the same 11 tick marks in the same locations (although the values on the scales varied) and data values were chosen so that all plotted points corresponded to a tick mark.

To classify the size of the effects we used the same procedure as used in the ACT-R model of Peebles (2013). We calculated the distance between the relevant plot points as the proportion, *p*, of the distance of the overall length of the y axis and then categorized the distance according the following scheme: “no” (*p* = 0), “very small” (0 < *p* < 0.2), “small” (0.2 ≤ *p* < 0.4), “moderate” (0.4 ≤ *p* < 0.6), “large” (0.6 ≤ *p* < 0.8), and “very large” (0.8 ≤ *p* ≤ 1.0). The resulting classifications of the eight graphs are shown in Table 1.

**Table 1:** **Size of main effects and interactions for the eight graph stimuli**.

**Graph**	**Main effect X**	**Main effect Z**	**Interaction**
1	Small	Large	No
2	Medium	Medium	Large
3	Large	Large	Small
4	Medium	No	Large
5	No	No	Large
6	No	Large	Medium
7	Very large	No	Small
8	No	Medium	Large

When matching data sets to graph content, care was taken to ensure that the effects depicted did not corresponded to commonly held assumptions about relationships between the variables (although this would be unlikely given the specialized nature of the graphs' subject matter).

The graphs were presented on A4 laminated cards and were drawn black on a light gray background with the legend variable levels colored green and blue. A portable digital audio recorder was used to record participants' speech as they carried out the experiment.

### 2.3. Procedure

The study was carried out in accordance with the ethical conduct recommendations of the British Psychological Society and was approved by the University of Huddersfield's School of Human and Health Sciences Research Ethics Committee. All subjects gave written informed consent in accordance with the Declaration of Helsinki.

Participants were seated at a table with eight bar or line graphs randomly ordered and face down in front of them and informed that their task was to try to understand each one as fully as possible while thinking aloud. In addition to concurrent verbalization during interpretation, participants were also asked to summarize the graph before proceeding to the next one.

During the experiment, if participants went quiet the experimenter encouraged them to keep talking. When participants had interpreted and summarized a graph, they were instructed to place the graph face down to one side and continue by turning over the next graph. Participants were not allowed to revisit graphs.

## 3. Results

### 3.1. Coding the verbal descriptions

A 2 × 2 experiment design results in three key potential effects: a main effect of the x axis IV, a main effect of the legend IV, and an interaction effect between the two. Data analysis involved coding whether each of the effects was identified and noting the time taken to interpret each graph. Audio recordings were transcribed prior to data coding with information identifying graph format being removed to ensure that coders were blind to graph format.

To meet the requirements for identification of main effects, participants had to state explicitly that there was an effect (e.g., from Figure 1F “There is a main effect of curing method”) or describe the effect of one of the IVs on a DV irrespective of the second IV (e.g., “Photocuring consistently produces a much higher fixtural strength than autocuring irrespective of cement type”).

To meet the requirements for identification of an interaction effect a participant had to state that there was an interaction effect (e.g., from Figure 1E “This shows a crossover interaction”) or describe how the effect of one of the IVs was moderated by the other (e.g., from Figure 1D “Treatment has a differential effect on CO_2_ uptake depending on plant type; when treatment is chilled, plant CO_2_ uptake is the same for both plant types but when treatment is non-chilled, plant CO_2_ uptake is lower in Quebec and higher in Mississippi.”

To illustrate the general speed and efficiency of many of the expert participants' interpretations, the example verbal protocol below is a verbatim transcription of a (not atypical) expert participant interpreting the line graph version of Figure 1G.

1. (Reads) “Glucose uptake as a function of fasting and relaxation training”
2. Alright, so we have…you're either fasting or you're not.
3. You have relaxation training or you don't.
4. And so…not fasting…er…
5. So there's a big effect of fasting.
6. Very little glucose uptake when you're not fasting.
7. And lots of glucose uptake when you are fasting.
8. And a comparatively small effect of relaxation training.
9. That actually interacts with fasting.

The protocol (which lasted 43 s) reveals the speed with which the variables and their levels are established and the key relationships within the data identified. Accuracy is not always perfect however; in addition to correctly identifying the main effect of the x variable and the interaction between the two IVs, the participant also incorrectly states that there is a (small) main effect of the legend variable.

The verbal protocols were coded by the second author and a sample of randomly selected codings (approximately 15% from each graph type) was independently scored by the first author. The level of agreement between the two coders was 96% for the bar graphs and 92% for the line graphs. When disagreements were found the raters came to a consensus as to the correct code.

### 3.2. Identification of effects

**Figure 2:**
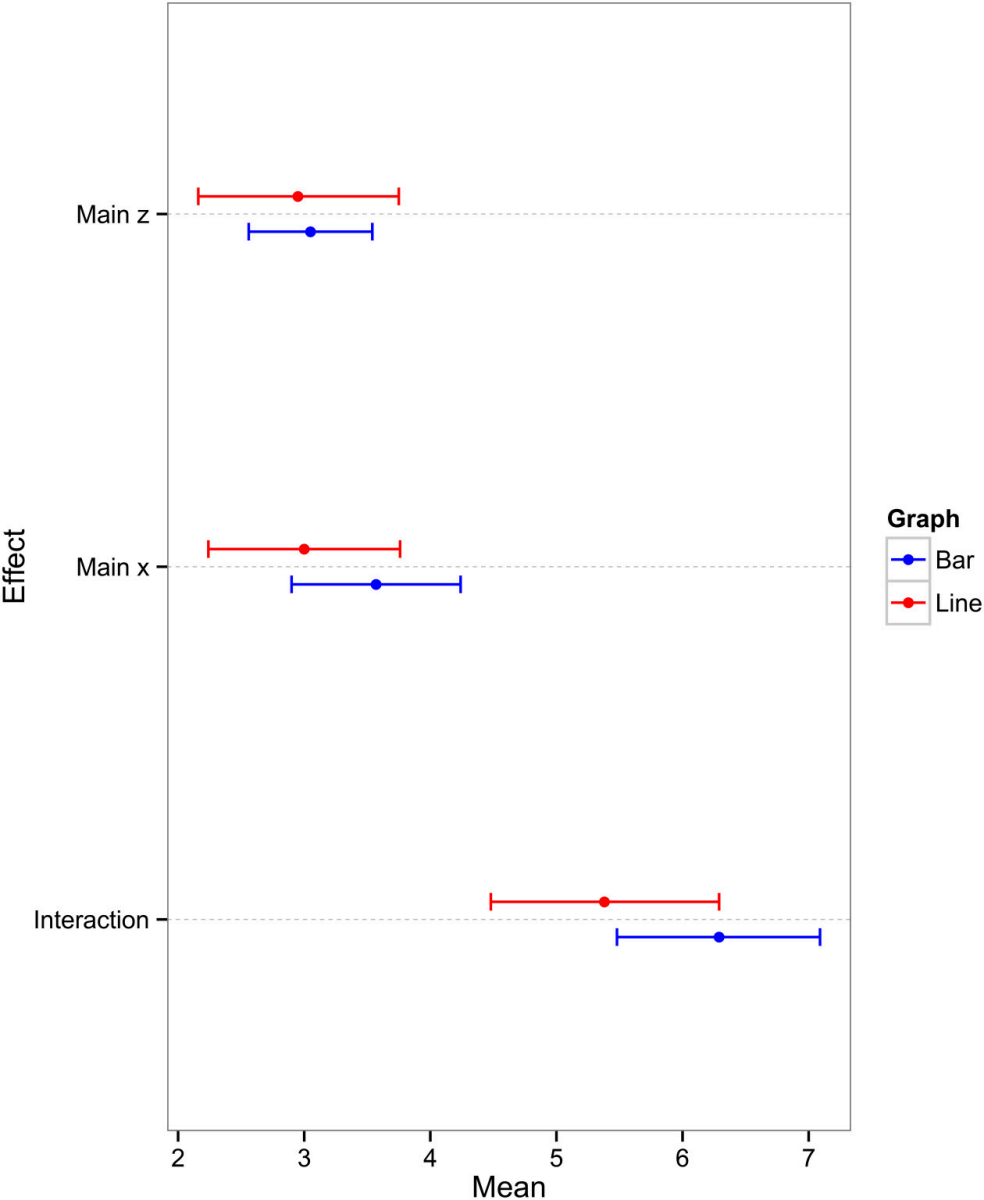
**Mean number of main effect x, main effect z, and interaction descriptions (with 95% confidence intervals) for the two graph conditions**.

Our initial analysis sought to determine whether experts' identification of main and interaction effects was affected by graph format. Figure 2 shows the mean number of identifications of the main effect of the x axis IV (henceforth referred to as “main effect x”), the main effect of the legend IV (henceforth referred to as “main effect z”), and interaction effect as a function of graph format.

Three independent sample *t*-tests revealed that graph format had no significant effect on participants' ability to identify the main effect x [*t*_(40)_ = 1.183, *p* = 0.246, *d* = 0.36], main effect z [*t*_(40)_ = 0.21, *p* = 0.832, *d* = 0.07], or interaction effect [*t*_(40)_ = 1.56, *p* = 0.127, *d* = 0.48]. The effect sizes vary from very small for main effect z to approaching medium for the interaction effect. In all cases, the pattern of responses was in favor of the bar graph condition but, in general, the results indicate that any bottom-up or top-down effects that may exist are not strong enough to bias experts' interpretations significantly in favor of one graph format over another. The present study therefore has not detected any effect of graph format on experts' ability to identify the key relationships in the data.

Another measure of the effect of graph format on performance is task completion time because this may indicate differences in interpretation strategy. A *t*-test on the mean task completion time for bar graphs (1 min, 25 s) and line graphs (1 min, 11 s) showed that this was not the case however [*t*_(29.783)_ = 1.077, *p* = 0.290, *d* = 0.3].

### 3.3. Main effect/interaction identification order

Although graph format does not lead to significant differences in the number of effects and interactions identified or the time taken to interpret a graph, it may be the case that the format of the graph affects the processes by which experts interpret them. For example, Shah and Carpenter (1995) found that people's understanding of the x-y relationship in three-variable line graphs was more comprehensive than their understanding of the z-y relationship due to the action of Gestalt processes whereas Peebles and Ali (2009) found the reverse effect in bar graphs. This typically leads to users focusing initially on the legend variable in line graphs and the x axis variable in bar graphs.

If expert users are susceptible to the same visual influences as novices, then it could be expected that they would be more likely to identify the main effect of the legend first in the line graph but the x axis main effect first in the bar graphs. Alternatively, experts' well-practiced strategies may override any such influences. To determine between these two hypotheses, we took trials where participants identified both main effects (20% of line graph trials and 23% of bar graph trials) and recorded which main effect was identified first.

The proportions of users selecting the x main effect before the z main effect was roughly equal between graph formats (line = 45.5%, bar = 44.7%) as was the case for the alternative order (line = 54.5% bar = 55.3%), indicating that, in contrast to novice users, experts are unaffected by Gestalt processes in this regard.

The two graph formats also differ in terms of the perceptual cues they provide to indicate the existence of an interaction. Line graphs provide a salient perceptual cue (cross pattern or non parallel lines) which is not as salient in bar graphs (Pinker, 1990; Kosslyn, 2006). In addition, there may be an expectation effect—experts may be influenced by their knowledge that line graphs are most often used to represent interactions and may therefore be primed to look for them (Shah and Freedman, 2009).

If this is the case, it could be expected that experts will identify interaction effects first in line graphs but main effects first in bar graphs. To test this, we took trials where participants identified both a main effect and an interaction (21% of line graph trials and 26% of bar graph trials) and recorded which one they identified first.

As with the previous analyses, there was no significant difference in the order of interaction and main effect identification between graph format conditions. The proportions of people selecting a main effect before the interaction effect was roughly equal between graph formats (line = 47%, bar = 50%) as was the case for the alternative order (line = 53%, bar = 50%). This shows that experts are influenced neither by an expectation that certain effects will be present in particular formats nor the more salient perceptual line graph cue indicating an interaction effect.

### 3.4. Interaction identification

Although we have found no differences in the patterns of identification due to Gestalt principles, user expectations, or different visual cues, the different perceptual cues in the two graphs may result in different patterns of inference to establish the existence of an interaction effect in bar graphs compared to line graphs. Specifically, interaction identification in line graphs may be triggered by the rapid identification of a salient pattern such as a cross and parallel lines [as assumed in the ACT-R model (Peebles, 2013)] whereas in bar graphs this pattern recognition process may not be as prevalent or influential.

To determine whether this is the case, we counted whether experts described the nature of the interaction prior to identifying the interaction effect in bar graphs and vice versa in line graphs. An example verbal protocol illustrating the first case recorded from a participant using the bar graph version of the graph in Figure 1B is presented below.

1. (Reads) “Maize yield as a function of plant density and nitrogen level”
2. When plant density is compact maize yield is higher.
3. Otherwise it's the same in all other conditions.
4. So it's an interaction between nitrogen level and plant density.

In contrast, an example verbal protocol illustrating the latter case recorded from a participant using the line graph in Figure 1E is listed below.

1. (Reads) “Cutting tool wear as a function of rock type and diamond type”
2. Straight away I see an interaction.
3. The effect of this factor is opposite depending on the rock type conditions.
4. If you have bead diamond type cutting tool wear is highest under limestone whereas bead under granite condition cutting tool wear is lower.
5. Bead works best in limestone and worse in granite.
6. In the wire it's the opposite trend. Cutting tool wear is lower in limestone and much higher in the granite.
7. Definite interaction. The other thing is the effect is very consistent; the two higher bars are 8 and the lower ones are at 5.
8. My summary is that if you're cutting limestone you want a bead type cutter, if it's granite then wire.

Only trials where participants described both the nature of the interaction and stated explicitly the existence of the interaction were included in the analysis. This amounted to 27% of line graph trials and 32% of bar graph trials. The proportion of participants who explicitly identified the interaction before going on to describe the nature of the effect was significantly larger in the line graph condition (80%) than in the bar graph condition, (54%, χ^2^ = 15.287, *df* = 1, *p* < 0.001). Analysis of the verbal protocols revealed that expert line graph users predominantly state the interaction immediately and then continue to describe the nature of the interaction whereas expert bar graph users would be equally likely to ascertain the nature of the relationship between the variables through a process of interrogation and reasoning followed by an explicit identification of the interaction.

Explaining this variance in behavior in terms of experts' different expectations is implausible as the previous process analysis found no differences in preference for identification of main effect and interaction order between the graph formats. The more convincing explanation in our view is that this observation is due to the bottom up influence of the salient patterns available in line graphs. It is important to note that this process difference does not result in a more superficial interpretation in the line graph condition; the richness of the descriptions was the same, just in a different order.

### 3.5. The influence of effect size

The analyses above demonstrated that graph format has no significant effect on the number of main or interaction effects identified by experts or the order in which they are interpreted. They have also provided no evidence that expectation has an influence upon the patterns and processes of experts' interpretations. We identified a third possible influence on expert interpretation however that may emerge from the relative sizes of the main and interaction effects in a particular data set.

To discover whether this factor determined the relative salience of effects (and thereby the order in which experts interpreted them) we took the distance values between plot points used to classify the effect sizes shown in Table 1 and tested whether these numerical values correlated with the order in which the effects were identified^2^. The analysis revealed a significant positive relationship between effect size and identification order—the larger the effect size, the greater the likelihood that the effect would be identified first, in both line [*r*_(21)_ = 0.647, *p* < 0.001] and bar [*r*_(21)_ = 0.730, *p* < 0.001] graphs.

## 4. Discussion

This study was designed to achieve three research goals related to issues concerning the nature of—and influences upon—expert comprehension performance. The first aim was to provide evidence that would allow us to adjudicate between two contrasting hypotheses concerning the relationship between levels of graphical literacy and the effect of graph format on interpretation. One hypothesis is that high levels of graphicacy will result in a reduction in the effect of graph format due to the increased ability to identify and mentally manipulate relevant information in the graph and generate appropriate inferences irrespective of the graphical features used to represent it (e.g., Pinker, 1990). The alternative hypothesis is that increases in graphicacy will result in an increase in the effect of graph format because graphicacy consists, at least in part, of a set of expectations and biases for different graph formats regarding their specific functions and properties (e.g., Zacks and Tversky, 1999; Shah and Freedman, 2009).

Although there was some evidence of expert expectation (a couple of participants commented that the bar graphs they were using should have been line graphs), the results of our experiment showed that whatever expectations some participants may have had, they had no significant effect on their interpretations. In fact the findings provide strong support for the former proposition by showing that experts' interpretations are, to all intents and purposes, identical for the two graph formats. There were no significant differences in the number of main effects or interactions that expert users were able to identify, nor in the time taken to identify them, related to the format of the graph (as indicated by the very small effect sizes).

The second aim of the study was to determine whether the processes or strategies by which experts achieve their interpretations using the two graphs differed in any significant way. Specifically we aimed to ascertain whether graph format affected the order in which experts interpreted the graph. In contrast to previous studies which have revealed a systematic interpretation order of legend variable followed by x axis variable in line graphs (Shah and Carpenter, 1995) and the opposite order in bar graphs (Peebles and Ali, 2009), experts in this study exhibited no such patterns of behavior, either in relation to the two main effects or in relation to the interaction and the main effects.

In addition, we sought to determine whether line graphs were more likely to result in a faster identification of certain relationships due to pattern recognition processes as argued by Kosslyn (2006). The results did support the hypothesis by showing that the graphical features of the line graphs did result in a more rapid identification of interactions than the bar graphs. More specifically, the verbal protocols suggested that participants in the line graph condition were indeed using pattern recognition processes to identify relationships in the data.

Finally, the experiment was conducted to determine whether the strategies that experts used to interpret data in these graphs were influenced by the relative effect sizes in the data and, if so, whether this differed between the graph conditions (perhaps as a result of differences in visual salience of the patterns formed by the graphical features in the two graph formats). The results revealed that experts are indeed sensitive to effect size and tended to identify large effects more rapidly than smaller effects, whichever graph format they used.

To summarize these results, while it does seem that experts are able to use the patterns in line graphs to more rapidly identify interactions, there is no overall benefit for experts of using line graphs over bar graphs. Although expert bar graph users may sometimes arrive at their interpretations via a different route, they take the same time and are no less likely to generate a full, correct analysis of the data than if they were to use a line graph.

This reveals that experts' greater experience allows them to ignore or override the pitfalls produced by Gestalt grouping processes in line graphs that novice users fall foul of (Peebles and Ali, 2009; Ali and Peebles, 2011, 2013) but does not result in experts constructing a set of expectations about the functions and properties of bar and line graphs that biases them detrimentally. Set in the broader context of the distinction between informational and computational equivalence of representations (Larkin and Simon, 1987), the experiment demonstrates how experts' knowledge of the possible relationships to look for in the data and the patterns that indicate them guides their search and reduces the effects of computational inequivalences and procedural constraints imposed by graphical format.

Taken together, these findings have a number of important implications for the presentation of data of this form, in particular regarding the question of which might be the best format to employ for the most widespread use (i.e., for both novice and expert users). Currently line graphs are used more often then bar graphs. A survey of graph use in a wide range of psychology textbooks by Peden and Hausmann (2000) showed that 85% of all data graphs in textbooks were either line graphs or bar graphs but that line graphs (64%) were approximately three times more common than bar graphs (21%). A similar but more recent survey which we carried out (Ali and Peebles, 2013) revealed that in leading experimental psychology journals, there was a slight preference for line graphs (54%) over bar graphs (46%) but a more pronounced preference in popular psychology textbooks; line graphs were favored 20% more than bar graphs.

In our previous work (Peebles and Ali, 2009; Ali and Peebles, 2013) however, we demonstrated that non-expert users performed significantly worse with line graphs compared to equivalent bar graphs and recommended that bar graphs (or an enhanced line graph that we designed) should be employed in cases where the aim is for accurate interpretation for a general audience of both novice and expert users.

Proponents of line graphs (e.g., Kosslyn, 2006) have argued, however, that the risk and costs of misinterpreting line graphs are outweighed by the benefit of lines for producing easily recognizable patterns that experts can associate with particular effects or interactions. The results of this study show however that although the patterns in line graphs are rapidly identified by experts, this does not lead to significantly better performance; experts are no less likely to identify key patterns in bar graphs as they are in line graphs, undermining the argument for the latter as a preferred representation.

The results of the study also have implications for models of expert graph comprehension. The current computational model of Peebles (2013) is based on a simple set of assumptions regarding pattern matching and memory retrieval which relate to the patterns formed by the x-y coordinates of the four data points (and are therefore not specific to any particular graph format). Currently the model does not take the size of effects into account when selecting a pattern to interpret. Instead patterns are selected at random.

The experiment has revealed that although experts can interpret bar and line graphs equally well, the processes by which they interpret them are affected by the format of the graph and also by the relative sizes of the effects in the data (irrespective of format). So while the data are broadly consistent with the assumptions of the model to the extent that experts do conduct an exhaustive search for the possible effects that may be present, a more accurate model will have to incorporate these additional factors. Once these factors are included, the resulting model will provide the most detailed and precise account of the knowledge and processes underlying expert comprehension performance for a widely used class of graphs in two formats.

Beyond the goal of extending the model to account for the full range of observed behavior with two graph formats lies the larger aim of developing and broadening the model to explain comprehension for a broader class of graphs. Interaction graphs embody a specific set of interpretive rules that are not shared by other more conventional graphs however because the data represent pairwise combinations of the IV levels so that the variables plotted are categorical, regardless of whether the underlying scale could be considered as continuous (e.g., hot/cold) or categorical (e.g., male/female).

The current model clearly identifies and characterizes these rules and distinguishes them from the knowledge and procedures that can be applied to other graphs. In so doing, the model simplifies the task of identifying graph-specific operators and forms a basis upon which to explore a range of comprehension models for other graph types.

In addition to furthering the development of formal models, the current work has also indicated further avenues for empirical investigation. Specifically, the significant influence of relative effect size found in the experiment suggests that expert interpretation is not immune from the constraints imposed by the visual salience of various patterns created by data. Future research on these factors will provide further valuable insights into the dynamic interplay between bottom-up and top-down processes on graph comprehension.

### Conflict of interest statement

The authors declare that the research was conducted in the absence of any commercial or financial relationships that could be construed as a potential conflict of interest.

## Footnotes

^1^Videos of the model in action can be found at http://youtu.be/qYY_No0i1Hc and http://youtu.be/IUU08KBmgMU.

^2^One graph (graph 2) was omitted from this analysis due to insufficient data.
